# P2X7 receptor involved in antitumor activity of atractylenolide I in human cervical cancer cells

**DOI:** 10.1007/s11302-022-09854-6

**Published:** 2022-03-02

**Authors:** Yue Han, Can Bai, Xi-Meng He, Qing-Ling Ren

**Affiliations:** 1 Department of Gynecology, The Affiliated Hospital of Nanjing University of Chinese Medicine, Nanjing, 210029 China; 2 Acupuncture & Chronobiology Key Laboratory of Sichuan Province, Chengdu University of Traditional Medicine, Chengdu, 610075 China

**Keywords:** Atractylenolide I, P2X7Rs, Hela cells, SiHa cells, Human cervical cancer cells

## Abstract

Atractylenolide I (Atr-I) was found to sensitize a variety of human cancer cells in previous studies. Purinergic P2X7R plays important role in different cancers. However, whether Atr-I could generate antitumor activity in human cervical cancer cells and P2X7R get involved in this effect remain unclear. In this study, Hela (HPV 18 +) and SiHa (HPV 16 +) cells were treated with different doses of Atr-I. The results indicated that agonist and antagonist of P2X7 receptors, BzATP and JNJ-47965567 (JNJ), could suppress the proliferation of Hela and SiHa cells. Atr-I demonstrated a considerable antitumor effect in both human cervical cancer cells in vitro. Atr-I combined with P2X7R agonist, BzATP, restored Atr-I-induced growth inhibition in Hela cells but not in SiHa cells. However, the combinatorial treatment of P2X7R antagonist JNJ and Atr-I has an additive effect on cell growth inhibition in SiHa cells rather than in Hela cells. It implied that P2X7R would get involved in the anti-human cervical cancer cells effect of Atr-I.

## Introduction

Cervical cancer is the fourth most common cause of morbidity and mortality among women with gynaecologic malignancies worldwide, and there were an estimated 600,000 new cases and 340,000 deaths in 2020 [1]. A large majority of cervical cancer (more than 95%) is due to the human papillomavirus (HPV) and HPV types 16 and 18 cause at least 70% of cervical cancers [2]. In the past few decades, despite the application of human papillomavirus (HPV) vaccine has been proven to dramatically reduce the risk of cervical cancer, the fact which cannot be neglected revealed that a high proportion of cervical cancer cases were clinically diagnosed. The surgical or radiotherapeutic-treated cases still suffer from recurrence or metastasis, which give rise to poor clinical outcomes and prognosis [3–5]. It is still a big challenge to find out new promising target or potential new drug for the treatment of cervical cancer [6, 7].

**Fig. 1:**
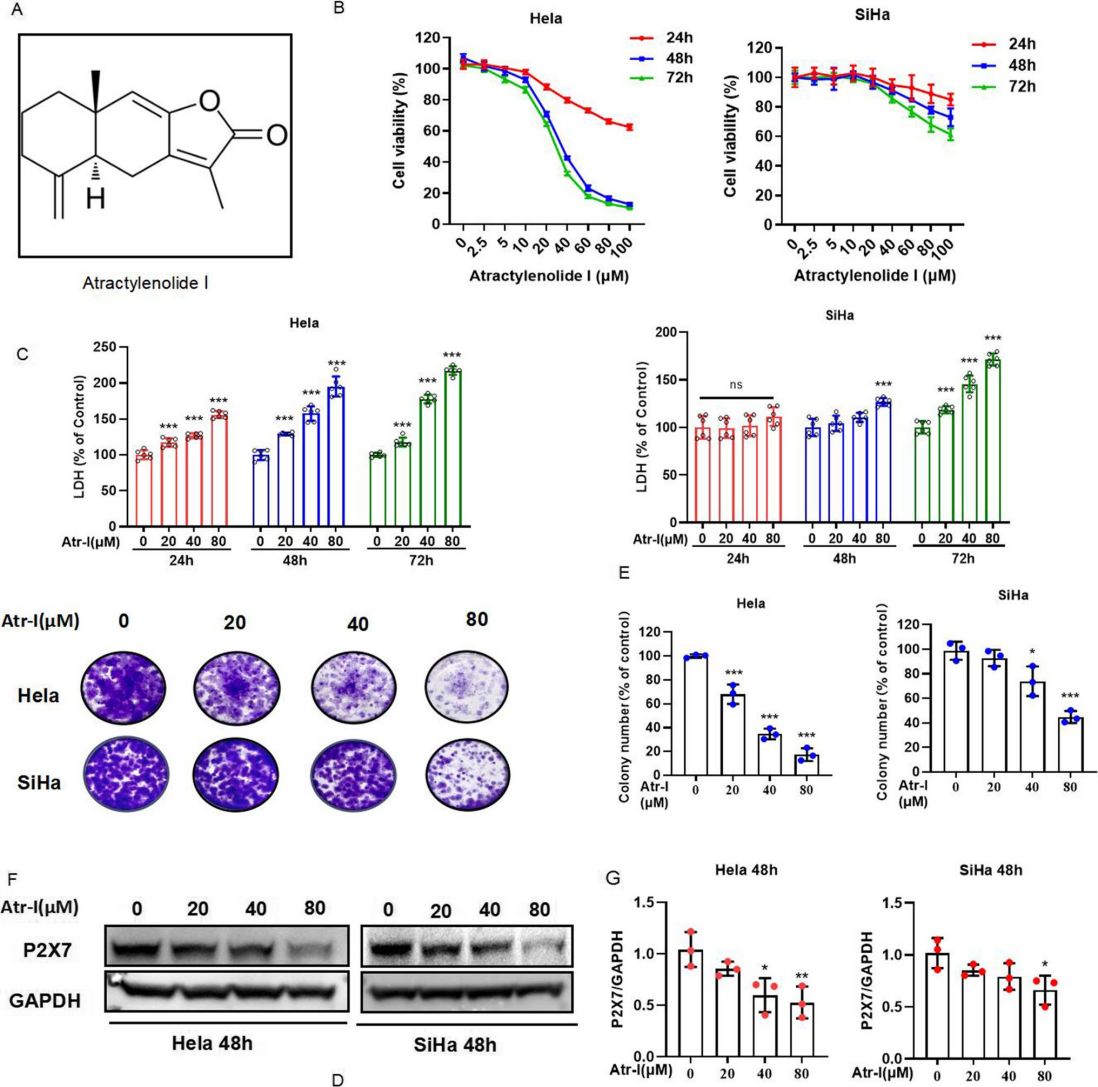
Atractylenolide I inhibits human cervical cancer cells’ proliferation in vitro. (**A**) Chemical structure of atractylenolide I. (**B**) The CCK8 assay of Hela cells and SiHa cells treated with the designed concentration of atractylenolide I for 24 h, 48 h, and 72 h. (**C**) LDH release assay in cells after exposure to increased atractylenolide I concentration for 24 h, 48 h, and 72 h. The colony formation assay of Hela cells and SiHa cells incubated with the increased atractylenolide I concentration for 2 weeks. The characteristic images (**D**) and the quantification of colonies (**E**). (**F**) The protein of P2X7R in Hela cells and SiHa cells was detected by Western blot after 48 h of the treatment with atractylenolide I and the quantification of the protein of P2X7R (**G**). All data are means ± SD. *p < 0.05, **p < 0.01,***p < 0.001 as compared with the atractylenolide I-untreated group

In recent years, purinergic signaling has been recognized to be a promising target in a variety of diseases in the whole body [8–10] since it was proposed by Burnstock in 1972 [11]. In the purinergic system, growing evidence supported that purinergic P2X7Rs play important role in different cancers, including lung cancer, colorectal cancer, breast cancer, and acute myeloid leukemia [12–19]. Apart from the strategy targeting the P2X7 ion channel, from antibodies and nanobodies to small-molecule drugs, which has been proved to improve the outcomes in models of cancer [20–24], the nature product is also taken into account for the development of potential anti-cancer drug to target purinergic receptors [25, 26]. Atractylenolide I (Atr-I, Fig. 1A), a natural product extracted from *Atractylodes macrocephala* Koidz, was found to sensitize human colorectal cancer cells, ovarian cancer cells, breast cancer, gastric cancer, and bladder cancer cells in previous studies [27–34]. However, whether Atr-I would be able to raise antitumor activity in human cervical cancer or P2X7 receptors would be involved in the antitumor activity of Atr-I remains unclear. Hence, this study aims to obtain the in vitro evidence of antitumor activity and the role of P2X7 receptors in Atr-I-treated human cervical cancer cells.

## Materials and methods

### Cell culture

Human cervical cancer cell lines (Hela, HPV 18 + ; SiHa, HPV 16 +) were obtained from the ATCC and cultured in DMEM supplemented with 10% fetal bovine serum (Thermo Fisher Scientific, USA), 100U/ml penicillin (Thermo Fisher Scientific, USA), and 100U/ml streptomycin (Thermo Fisher Scientific, USA), at 37 °C in 5% CO_2_.

### Antibodies and reagents

Antibodies were purchased from Proteintech (GAPDH Monoclonal antibody 60,004–1-Ig, HRP-conjugated Affinipure Goat Anti-Rabbit IgG (H + L) SA00001-2, HRP-conjugated Affinipure Goat Anti-Mouse IgG(H + L) SA00001-1) or Abcam (P2X7R ab93354, UK). The specific agonist to P2X7R, 2′(3′)-O-(4-benzoylbenzoyl) adenosine 5′-triphosphate (BzATP, B6396) was purchased from Sigma. The selective P2X7 antagonist JNJ-47965567 (JNJ) was purchased from Tocris Bioscience (Bristol, UK). Atr-I (73,069–13-3) was purchased from Herbpurify (Chengdu, China).

### Cell proliferation inhibition test

Cell Counting Kit-8 (CCK-8, AbMole, USA) and colony formation assays were used to detect cell proliferation. Briefly, the cells used for CCK-8 assay were inoculated (1 × 10^6^ cells/ml) into a 96-well plate, followed by the addition of 100 µl of cell suspension to each well and received the designed concentrations of drug treatment. The detailed procedures have been described by Bai et al. [35]. The cells for colony formation assay were cultured in 24-well plates (800 cells/well) and incubated with the designed treatments. After 2 weeks, the 4% paraformaldehyde was adopted for fixation and crystal violet was used for staining. The visible colonies were calculated applying ImageJ software.

### Western blot analysis

Cells were lysed in ice with RIPA buffer (150 mmol/l Tris–HCl pH 7.0, 150 mmol/l NaCl, 1% NP-40, 1% sodium deoxycholate, 0.1%SDS) containing with protease inhibitor cocktail (Roche, 4,693,159,001) for 30 min. The Bio-Rad protein assay was used to quantify the lysates. The protein samples were subjected to NuPAGE™ 10% Bis–Tris Protein Gels (Invitrogen, NP0302BOX) and probed with the indicated antibodies to visualize the protein levels using a ChemiScope 6000 Touch.

### Lactate dehydrogenase release assay

Lactate dehydrogenase (LDH) test kit (Beyotime Biotechnology, Shanghai, China) was used to assess the cytotoxicity of atractylenolide I and in combination with or without 100 μM BzATP or 20 μM JNJ. Cells were cultured in 96-well plates (6 × 10^3^cells/well). After treatment with the designed concentrations of drugs, cell culture supernatant was transferred to the new 96-well plate for LDH analysis.

### Flow cytometry

The ratio of apoptotic cells was measured with an Annexin V-AF647/PI Apoptosis Kit (E-CK-A213; Elabscience biotechnology, Wuhan, China) according to the manufacturer’s instructions. Cells were harvested and washed twice with PBS and then resuspended in 500 μl binding buffer. After adding 5 µl annexinV-AF647 and 5 μl PI into the cell suspension, respectively, at least 20,000 live cells were analyzed on a FACSCalibur flow cytometer (BD Biosciences, San Jose, CA, USA). Data were analyzed by using FlowJo software (FlowJo, Ashland, OR, USA).

### Statistical analysis

All experiments were performed at least three times independently. All statistical analysis and graphics were performed using GraphPad Prism 7.0 Software (GraphPad Software Inc., San Diego, CA). For two-group comparisons, Student’s two-tailed *t*-test was used. For multiple group comparisons, one-way ANOVA was used. A value of *P* < 0.05 was considered statistically significant.

## Results

### Atractylenolide I represses human cervical cancer cells’ growthin vitro

To evaluate whether Atr-I exhibits an antitumor effect against human cervical cancer cells, Hela cells and SiHa cells were treated with different doses of Atr-I. CCK8 assay showed that Atr-I markedly inhibited the growth of Hela cells and SiHa cells in a dose- and time-dependent manner. Notably, Hela cells were highly sensitive to Atr-I, whereas SiHa cells demonstrated higher tolerance to Atr-I (Fig. 1B). Consistently, the proliferation of Hela cells was significantly inhibited than that of SiHa cells in response to Atr-I treatment, as evidenced by reduced colony formation (Fig. 1D, E). Then, we performed LDH release assay and found that Atr-I damaged the integrity of the plasma membrane, which was most obvious at 72 h after treatment (Fig. 1C). Taken together, these results suggest that Atr-I demonstrate a considerable antitumor effect in human cervical cancer cells in vitro.

### The antitumor activity of atractylenolide I involves the P2X7 receptor in human cervical cancer cells

**Fig. 2:**
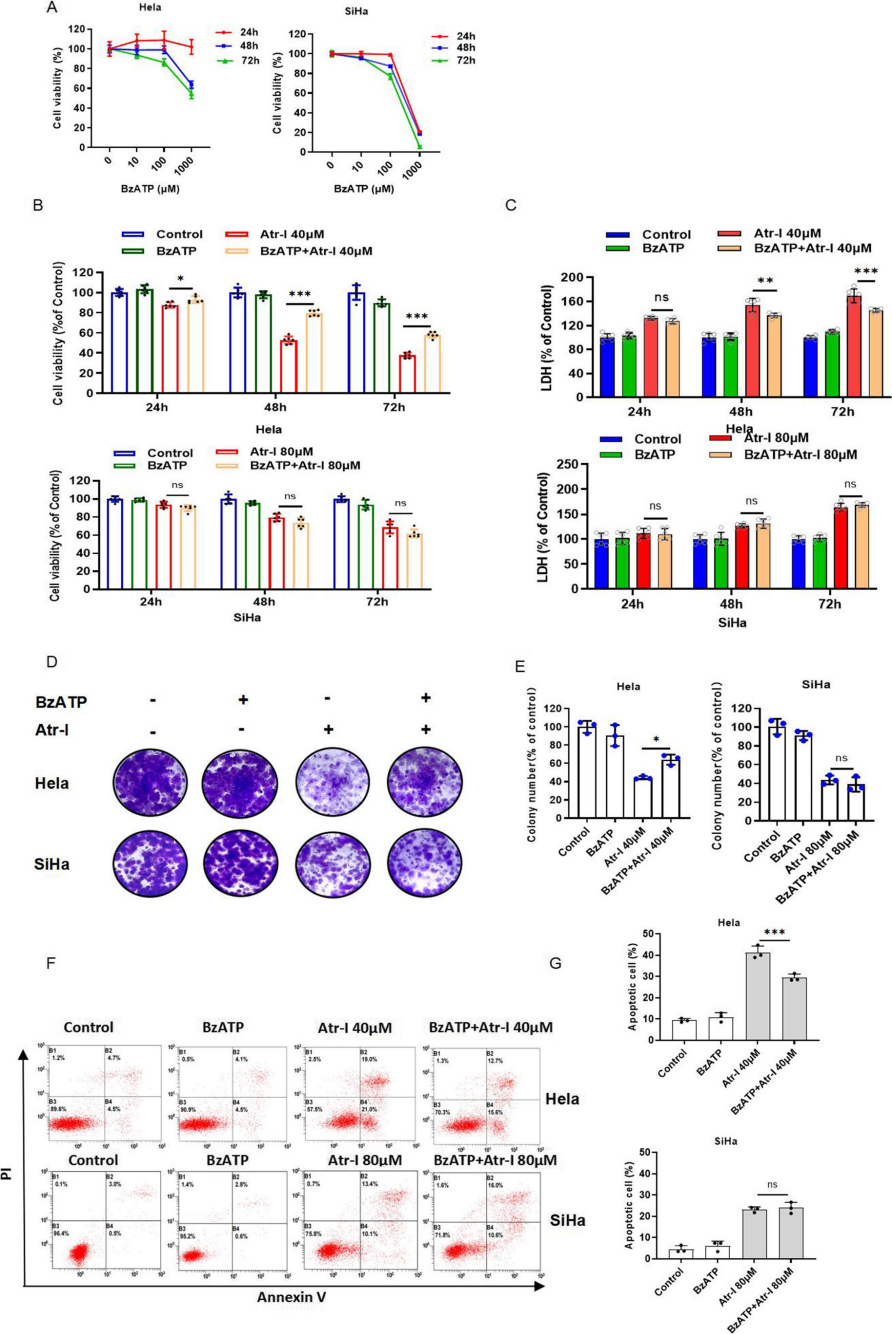
Activation of P2X7R affects cervical carcinoma cells’ proliferation. (**A**) The CCK8 assay of Hela cells and SiHa cells treated with the designed concentration of BzATP for 24 h, 48 h, and 72 h. (**B**) CCK8 assay of Hela cells and SiHa cells indicated the presence or absence of atractylenolide I and in combination with or without 100 μM BzATP for 24 h, 48 h, and 72 h. (**C**) LDH release assay in cells treated as in B. The colony formation assay in cells incubated under these same conditions for 2 weeks. The characteristic images (**D**) and the quantification of colonies (**E**). (**F**, **G**) Hela cells and SiHa cells treated with atractylenolide I or 100 μM BzATP alone or their mixture for 48 h were subjected to flow cytometry analysis. All data are means ± SD. *p < 0.05, **p < 0.01, ***p < 0.001

**Fig. 3:**
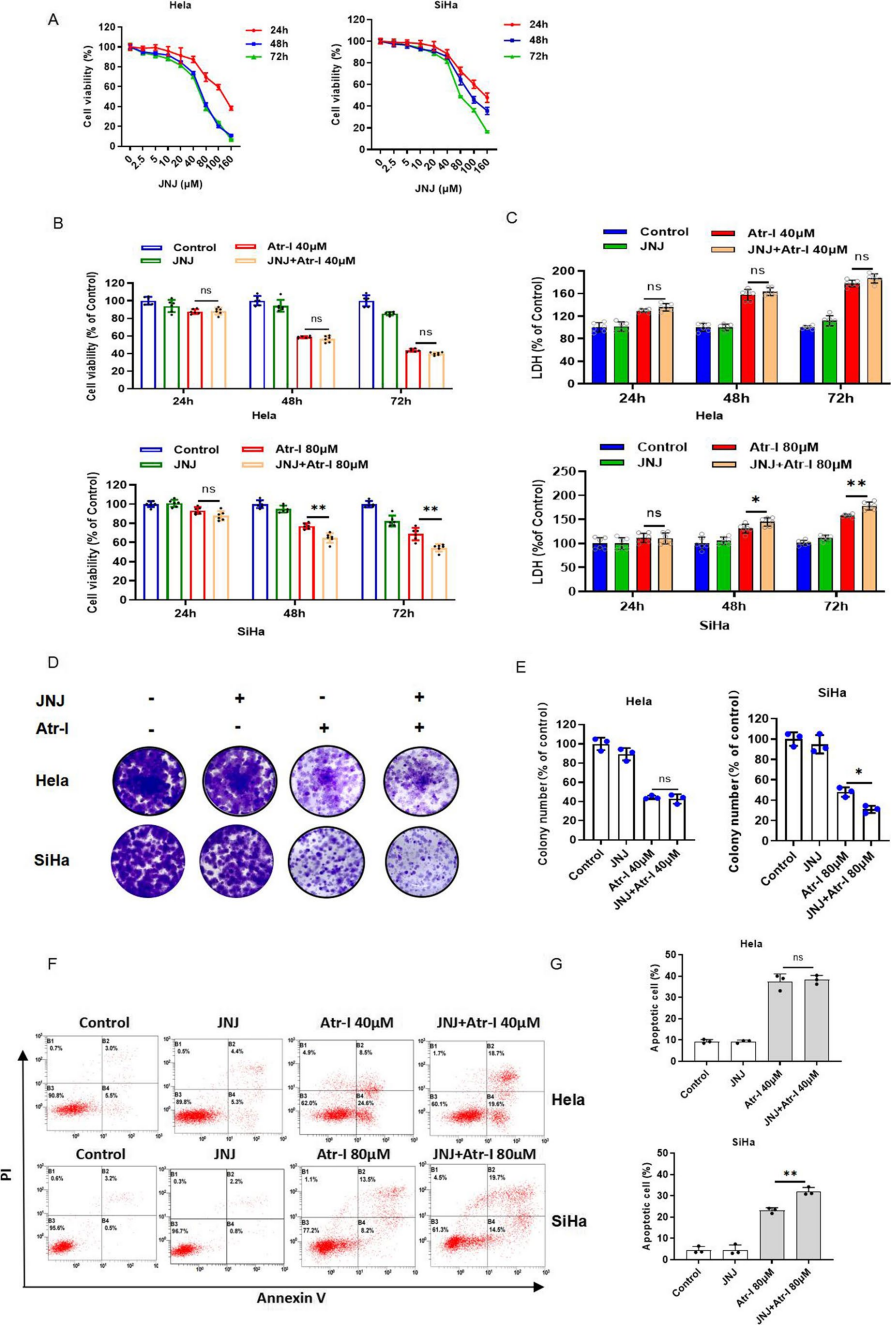
Inhibition of P2X7R affects cervical carcinoma cells’ proliferation. (**A**) The CCK8 assay of Hela cells and SiHa cells treated with the designed concentration of JNJ for 24 h, 48 h, and 72 h. (**B**) CCK8 assay of Hela cells and SiHa cells indicated the presence or absence of atractylenolide I and in combination with or without 20 μM JNJ for 24 h, 48 h, and 72 h. (**C**) LDH release assay in cells treated as in B. The colony formation assay in cells incubated under these same conditions for 2 weeks. The characteristic images (**D**) and the quantification of colonies (**E**). (**F**, **G**) Hela cells and SiHa cells treated with atractylenolide I or 20 μM JNJ alone or their mixture for 48 h were subjected to flow cytometry analysis. All data are means ± SD. *p < 0.05, **p < 0.01, ***p < 0.001

Next, we intended to evaluate whether P2X7 receptor was involved in the anti-human cervical cancer cells’ effect of Atr-I. Firstly, the western blot analysis indicated that the decreased expression of P2X7 receptor protein was observed in Atr-I-treated Hela cells and SiHa cells (Fig. 1F, G). And then, CCK8 assay showed that BzATP, the selective agonist of P2X7 receptor, and JNJ, a selective small-molecule antagonist of P2X7 receptor, markedly inhibited the growth of Hela cells and SiHa cells with a dose- and time-dependent manner (Fig. 2A, Fig. 3A). Interestingly, SiHa cells are more sensitive to BzATP. When human cervical cancer cells were treated with BzATP or JNJ and Atr-I together, the data indicated that BzATP partially restored Atr-I-induced growth inhibition in Hela cells, but it was hardly affected in SiHa cells (Fig. 2B). Consistently, the LDH release of Hela cells was significantly inhibited in response to combinational treatment of BzATP (Fig. 2C), as evidenced by increased colony formation (Fig. 2D, E). Whereas the combinatorial treatment of JNJ and Atr-I has an additive effect on cell growth inhibition in SiHa and Hela cells (Fig. 3B). Consistently, the LDH release of SiHa cells was significantly elevated in response to combinational treatment of JNJ (Fig. 3C) after 48 h, as evidenced by reduced colony formation (Fig. 3D, E). Finally, the data from flow cytometry presented that BzATP partially restored Atr-I-induced apotosis in Hela cells but not in SiHa cells (Fig. 2F, G). Whereas the combinatorial treatment of JNJ and Atr-I, JNJ partially promoted Atr-I-induced apotosis in SiHa cells but not in Hela cells (Fig. 3F, G). Taken together, these results implied that the P2X7 receptor was involved in the anti-human cervical cancer cells effect of Atr-I with different roles in HeLa cells and SiHa cells.

## Discussion

In this study, we firstly found that Atr-I generated a considerable antitumor effect in 2 different HPV phenotypes of human cervical cancer cell lines (Hela, HPV 18 + ; SiHa, HPV 16 +) in vitro. It supports that Atr-I would be a potential promising anti-cancer compound for the treatment of human cervical cancer, especially caused by HPV 18 and HPV 16, as well as colorectal, ovarian, breast, gastric, and bladder cancers [27–34]. However, due to the fact that in the current study Hela cells were highly sensitive to Atr-I, whereas SiHa cells demonstrated higher tolerance to Atra-I, it implied that we cannot neglect that the different cell lines would be the reason to present different responses to Atr-I treatment. Additional cell lines of human cervical cancer, including Bu25TK, CaSki, MS751, C33A, Me180, and HT-3, or in vivo data is needed to confirm the antitumor effect of Atr-I.

Based on current data, it seems that the role of purinergic P2X7R in the antitumor effect of Atr-I is diversity. The reduced protein expression of P2X7 receptor in both Hela and SiHa cells was observed in this study. It implied that P2X7R might be able to get involved in the antitumor mechanism of Atr-I in 2 different human cervical cell lines. In Hela cells, co-application of P2X7 agonist BzATP and Atr-I led to the decreased antitumor effect whereas no changes were found under the condition combination of P2X7 antagonist JNJ and Atr-I. It suggested that the antitumor effect of Atr-I in Hela cells with HPV 18 positive would be linked with P2X7 receptor. However, it would be another possibility in SiHa cells with HPV 16 positive. Co-administration of P2X7 antagonist JNJ rather than the agonist BzATP and Atr-I raised the antitumor effect although the single use of both agonist of P2X7 BzATP and the antagonist JNJ could generate antitumor effect in Hela and Siha cells. It could be proposed that the antitumor effect of Atr-I in Siha cells would be associated with purinergic P2X7 receptors and its downstream pathway (PI3K/Akt/GSK-3β, AKT, AMPK-PRAS-40-mTOR, NOD-like receptor containing a pyrin inflammasome, etc.) which is regarded as the underlying mechanism of P2X7 receptors in cancers [36–39].

It is worth pointing out that in our study both BzATP and JNJ have antitumor effect in Hela and SiHa cells. BzATP is the agonist to active P2X7 receptor, theoretically which would be harmful for the treatment of cancer in that increasing evidence demonstrated that suppression of high concentration of ATP in tumor microenvironment and the activation of P2X7 is recognized as a promising strategy for the treatment of different tumors [12, 18, 20, 38, 39]. However, ATP is considered a double-edged sword in cancer [40, 41] and BzATP could inhibit breast cancer cell migration [42]. It indicated that both the agonist (BzATP) and antagonist (JNJ) of P2X7 receptor would be useful to inhibit the proliferation of human cervical cancer. Maybe the reason would be related to the complicated regulation of downstream signaling pathways by P2X7, including the concentration and duration of agonist exposure, the interaction of P2X7 with other receptors, and the interaction with the actin cytoskeleton machinery [38, 39, 43–45]. On the other hand, co-administration of P2X7 antagonist JNJ rather than the agonist BzATP and Atr-I raised the antitumor effect, which might be derived from the complicated microenvironment of human cervical cancer. More comprehensive evidence would be pursued in the future design and research.

## Conclusions

This study demonstrated that both the agonist and antagonist of P2X7 receptors, BzATP and JNJ, could suppress the proliferation of Hela and SiHa cells. And the P2X7 receptor would get involved in the antitumor effect of Atr-I in the 2 different human cervical cancer cell lines (HPV 18 + in hela and HPV 16 + in SiHa) with different HPV phenotypes.

## Data Availability

The datasets generated during and/or analyzed during the current study are available from the corresponding author on reasonable request.
